# Supplementary material for: Causal Relationship between Aging and Anorexia Nervosa: A White-Matter-Microstructure-Mediated Mendelian Randomization Analysis
Source: Biomedicines. 2024 Aug 16;12(8):1874. doi: 10.3390/biomedicines12081874 (PMC11351342; doi:10.3390/biomedicines12081874)
Supplement: Supplementary file 1 [file biomedicines-12-01874-s001.zip › Supplementary Figure/Figure S4/Singe SNP plot of MR analysis for TL to ubm-b-1541.pdf]

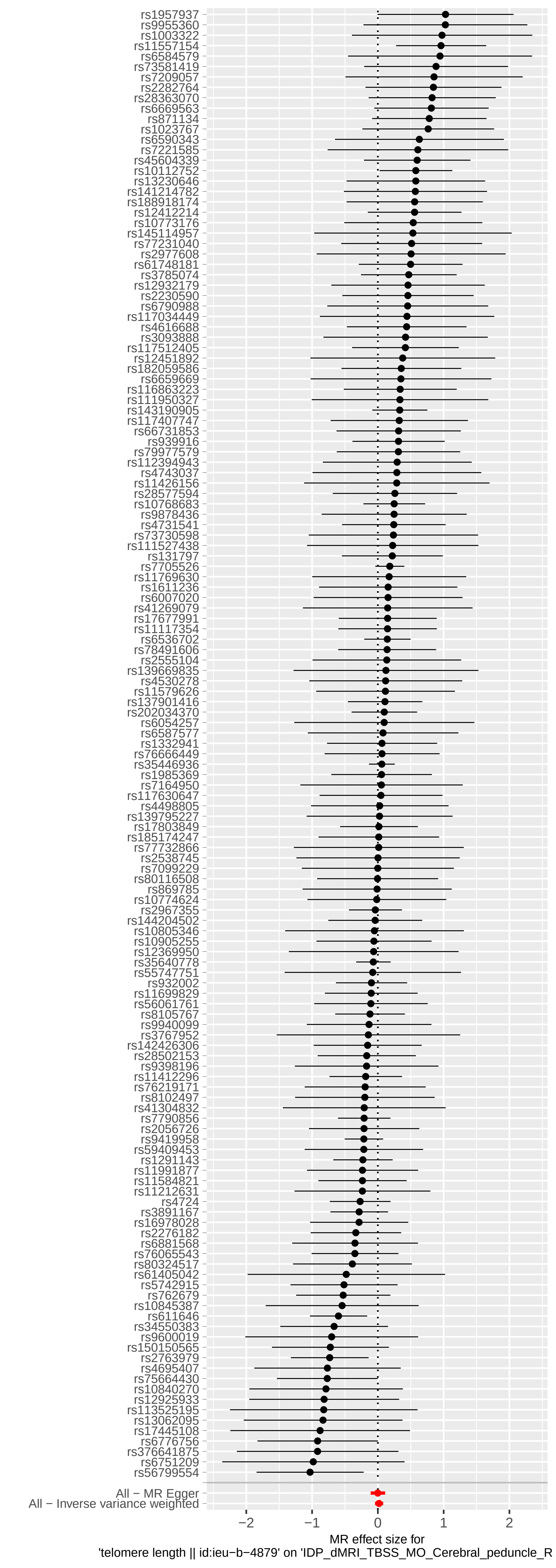
rs1957937
rs9955360
rs1003322
rs11557154
rs6584579
rs73581419
rs7209057
rs2282764
rs28363070
rs6669563
rs871134
rs1023767
rs6590343
rs7221585
rs45604339
rs10112752
rs13230646
rs141214782
rs188918174
rs12412214
rs10773176
rs145114957
rs77231040
rs2977608
rs61748181
rs3785074
rs12932179
rs2230590
rs6790988
rs117034449
rs4616688
rs3093888
rs117512405
rs12451892
rs182059586
rs6659669
rs116863223
rs111950327
rs143190905
rs117407747
rs66731853
rs939916
rs79977579
rs112394943
rs4743037
rs11426156
rs28577594
rs10768683
rs9878436
rs4731541
rs73730598
rs111527438
rs131797
rs7705526
rs11769630
rs1611236
rs6007020
rs41269079
rs17677991
rs11117354
rs6536702
rs78491606
rs2555104
rs139669835
rs4530278
rs11579626
rs137901416
rs202034370
rs6054257
rs6587577
rs1332941
rs76666449
rs35446936
rs1985369
rs7164950
rs117630647
rs4498805
rs139795227
rs17803849
rs185174247
rs77732866
rs2538745
rs7099229
rs80116508
rs869785
rs10774624
rs2967355
rs144204502
rs10805346
rs10905255
rs12369950
rs35640778
rs55747751
rs932002
rs11699829
rs56061761
rs8105767
rs9940099
rs3767952
rs142426306
rs28502153
rs9398196
rs11412296
rs76219171
rs8102497
rs41304832
rs7790856
rs2056726
rs9419958
rs59409453
rs1291143
rs11991877
rs11584821
rs11212631
rs4724
rs3891167
rs16978028
rs2276182
rs6881568
rs76065543
rs80324517
rs61405042
rs5742915
rs762679
rs10845387
rs611646
rs34550383
rs9600019
rs150150565
rs2763979
rs4695407
rs75664430
rs10840270
rs12925933
rs113525195
rs13062095
rs17445108
rs6776756
rs376641875
rs6751209
rs56799554
All − MR Egger
All − Inverse variance weighted
−2
−1
0
1
2
MR effect size for
'telomere length || id:ieu-b-4879' on 'IDP_dMRI_TBSS_MO_Cerebral_peduncle_R
